# Unlocking Ethiopia’s genomic landscape and its global significance: a call for inclusive genomics research

**DOI:** 10.1186/s40246-025-00878-8

**Published:** 2026-01-29

**Authors:** Sisay Teka Degechisa, Tesfaye B. Mersha

**Affiliations:** 1 Department of Medical Biochemistry, College of Health Sciences, Addis Ababa University, Addis Ababa, Ethiopia; 2 Department of Medical Laboratory Sciences, College of Medicine and Health Sciences, Arba Minch University, Arba Minch, Ethiopia; 3 Precision Pulmonary Medicine Research Program, Department of Medicine, Division of Pulmonary, Critical Care, and Sleep Medicine, Indiana University School of Medicine, 980 W. Walnut Street, Indianapolis, IN 46202 USA

**Keywords:** Ethiopia, Gene flow, Positive selection, High altitude, Adaptation, Lactase persistence, Sour taste perception, Energy metabolism, Genetic structure, Haplotype, African genome, Linguistic variation, Admixture, Precision medicine

## Abstract

Ethiopia, located at the intersection of Africa and Eurasia, is a hub of human genetic diversity and cultural richness. Its proximity to the Middle East has historically positioned it as a vital trade corridor connecting Asia, Europe, and Africa. Located along both the “out of Africa” and “back to Africa” human migration routes, Ethiopia has become one of the most genetically, ethnically, culturally and linguistically diverse countries in the world. This diversity is further shaped by adaptations to a wide range of environments, from the peaks of the Semien Mountains (4550 m or 14,928 feet above sea level) to the arid Danakil depression (100 m or 328 feet below sea level), both of which harbor rich fauna and flora. Despite its strategic location and rich genetic diversity, Ethiopian populations remain underrepresented in global genomics research. This review: (1) highlights key examples of genetic adaptations that shape the Ethiopian gene pool, including positive selection for high- and low-altitude adaptation, lactase persistence, UV exposure, disease resistance, sour taste perception, and metabolism, and (2) calls for genetics studies that incorporate the unique genetic evolutionary history of the Ethiopian population, which can generate important scientific insights. In the era of precision medicine, it is essential to include genetically diverse populations, such as Ethiopia’s, to ensure the advancement of clinical medicine for everyone.

## Introduction

### Background

Hominin fossil records and religious scripts indicated that Abyssinia, modern-day Ethiopia, is a key region in human origins and early civilization [1, 2]. Its role as a historical trade hub [3] attracted people from diverse backgrounds, fostering rich ethno-linguistic diversity, which in turn contributed to extensive gene flow, shaping a wide range of modern human genetic lineages [4–6]. Historically, ancient kingdoms such as Axumite and Zagwe drove demographic shifts and genetic admixture through empire expansion south into Africa and Arabia [6]. While earlier interpretations characterized the physical movement of northern Ethiopian highlanders as primarily influenced by Middle Eastern sources and southern populations by broader African groups [5, 7], this view is now considered oversimplified. Recent studies demonstrate that gene flow was complex and multidirectional, sweeping across both North-South and high-to-lowland gradients. Much of what was previously identified as “Middle Eastern gene flow” is more accurately understood as an ancestral component of ancient Cushitic or East African populations that later admixed with groups carrying Arabian-related gene flow [8–10], reinforcing the fundamentally African heritage of Ethiopian populations.

Ethiopia’s geographic location has historically enabled connections among people from diverse cultural, ethnic, and religious backgrounds across continents, facilitated by its proximity to the Middle East via the Red Sea [11]. This connectivity helped shape its role as a cultural and religious crossroads, further enriched by the Nile River, which served as a conduit to the Ethiopian highlands. Since ancient times, Ethiopia has been home to the three Abrahamic religions (Judaism, Christianity, and Islam), all of which originated in the Middle East. The region also provided as a refuge for those fleeing religious persecution, as legacy in both Christian and Muslim histories [12].

Recent Eurasian gene flow through the “back to Africa” migration influenced the genetic structure of the Ethiopian genome [13, 14]. For example, admixture analyses revealed that 40–50% of the genetic ancestry in Ethiopian populations speaking Semitic and Cushitic languages is shared with non-African populations [5]. Consistent with this phylogenetic analysis, Ethiopian Afro-asiatic-speaking groups, such as the Amhara and Dizi, display high proportions of Eurasian-related gene flow compared to many other Sub-Saharan African groups [15, 16]. Anecdotal evidence from recent participants of Ethiopian origin in the All of Us Research Program [17] indicated that approximately 60% of their genetic ancestry traced to Middle Eastern/North African descent, with the remainder linked to Central and East African populations, including Kenya, Uganda, the Congo Basin, and Angola (personal communication). This observation is consistent with published data showing that the Ethiopian population represents an intermediate between sub-Saharan African and Southwest Asian ancestries [5, 14, 18].

Ethiopia’s overall genetic diversity is also influenced by its vast environmental heterogeneity, which spans extreme variations in elevation from high-altitude (HA) regions (up to 4,550 m above sea level) to low-lying areas (down to 100 m below sea level) [19]. This diverse environment fosters local adaptation, contributing to a high degree of genetic variation. However, this diversity is not randomly distributed; instead, its structure is fundamentally governed by geographic proximity [18, 20]. Of note, certain observable genetic similarity among Ethiopians, despite their extensive cultural and ethnic diversity, is primarily driven by geographic proximity, where physical distance dictates genetic relatedness [18]. Similarly, a study conducted in Northern Kenya supports the idea that geographic proximity is a major determinant of genetic similarity among Kenyan populations [21]. This relationship is a classic example of isolation-by-distance and aligns with Tobler’s First Law of Geography, which states that “Everything is related to everything else, but near things are more related than distant things” [22]. The deep historical influence of geography is further evidenced by the decreasing ancestry related to the 4500-year-old Mota individual with spatial distance from its discovery site [14, 20]. Others reported that Ethiopian populations’ genetic structure is broadly correlates with linguistic affiliation, most clearly separating the major Afro-asiatic (Cushitic, Semitic, Omotic) and Nilo-Saharan families. However, the correlation pattern remains weak due to historical admixture among ethnic groups, migrations and ancient movements [5, 20]. Social strata could also serve as a mechanism for genetic divergence by enforcing isolation [18], particularly in marginalized minorities such as Ari blacksmiths who exhibit high genetic differentiation [20].

Ethiopia’s genomic landscape is defined by high intra-population genetic diversity resulting from its complex demographic history and multi-directional gene flow from other populations as a result of admixture. This cumulative history of multiple migration and admixture events, coupled with continuous adaptive selection pressure from local factors like altitude, generated the observed mosaic of distinct genetic profiles across Ethiopia’s diverse populations. In this review, we aim to (1) provide a comprehensive overview of genetic diversity, patterns of admixture, and adaptive selection among Ethiopian populations that contribute to positive selection, etc., and (2) highlight key examples and calls for genetics studies to unravel Ethiopia’s unique genetic landscape and its contribution to global human diversity. Understanding the genetic lineage of Ethiopian populations is critical not only for advancing knowledge of the Ethiopian population but also for translating genetic insights into improved disease risk prediction and precision medicine across diverse populations. This review may also serve as a useful starting point for scholarly discussions and future research across disciplines such as history, anthropology, sociology, and linguistics in the context of genetic diversity.

### Migration shaped Ethiopian genetic diversity

Following the emergence of *Homo sapiens* in Africa, humans spread across the globe, migrating through Arabia, into Eurasia, the Pacific, and eventually the Americas. These migration patterns laid the foundation of global genetic diversity [23]. Within Ethiopia, historical records, from Rossini’s 1928 account of ancient history (covering the 4th century to 1270), to the Early Solomonic period (1270–1527), and modern histories by Pankhurst and Zewdie (1895–1991), documented extensive internal migration across geographic boundaries [24–27]. For example, the early southward migration of Judeo-Christian highlanders has have played a role in the dispersal of populations as far south as the tip of Africa [28, 29], while the 16th -century northward expansion of the Oromo people introduced further genetic admixture and cultural exchange [29]. These movements fostered significant cultural and linguistic diversity within Ethiopia, with some southern populations sharing cultural traits with northern highlanders despite belonging to different linguistic families. This extended period of close contact and intermixing contributed to the development of languages with highly blended linguistic features, where the grammar or phonology of one language family significantly influenced the structural characteristics of another [30].

These complex migration patterns, both internal and external, spanning centuries, have profoundly shaped Ethiopia’s genetic diversity. The movements have fostered rich cultural and linguistic intermixing, resulting in a mosaic of genetic lineages that reflect Ethiopia’s role as a crossroads of civilizations. Despite this rich genome diversity, Ethiopian populations remain underrepresented in global genomics research [31], underscoring the urgent need for inclusive studies that capture the full spectrum of genetic variation. Such efforts will not only illuminate the evolutionary history of human populations but also enhance our understanding of disease susceptibility and resilience in diverse environments [5, 18, 20, 32–34].

### Ethiopia’s role as a gateway in shaping global human genetic diversity

**Fig. 1:**
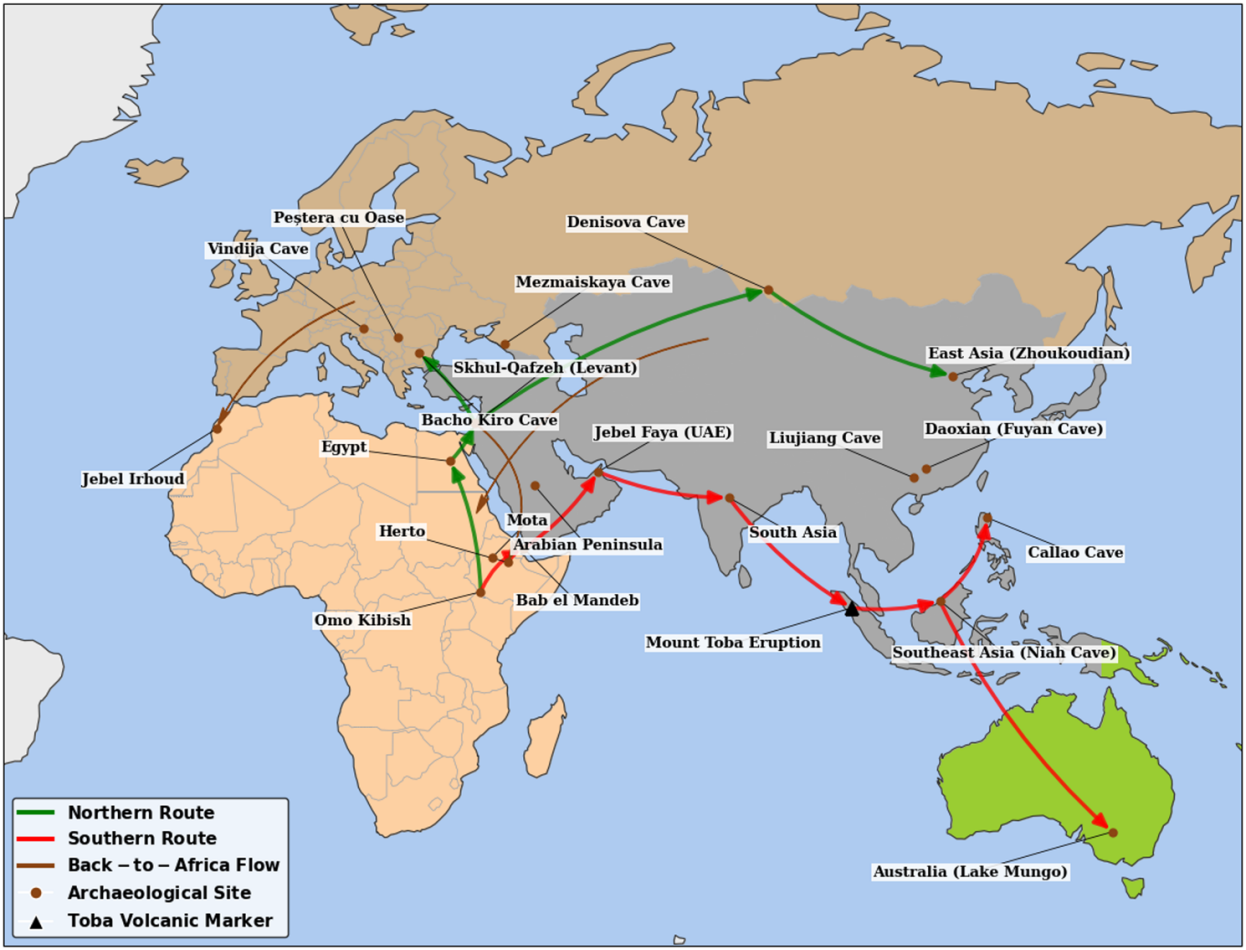
This map illustrates the dual-route dispersal of Anatomically Modern Humans (AMH) originating from core sites in Ethiopia, such as Omo Kibish and Herto. The Northern Route (green) traces the expansion from the Ethiopian highlands through the Nile Valley into the Levant and Europe, while the Southern Route (red) exits the Horn of Africa via the Bab el Mandeb toward Asia and Australia. The brown dots represent important archeological sites. The black triangle is a marker of volcanic eruption. Highlighting Jebel Irhoud on the western Moroccan border as a key early landmark, the map also identifies Back-to-Africa (brown) migrations representing later return flows from Eurasia into North and East Africa. This map was constructed via Google Colab [41] based on the map from [42]

While debates persist regarding the precise routes of early human dispersal, early theories proposed two main routes for “out of Africa” migration: a northern route through Egypt and Sinai, and a southern path through Ethiopia, the Bab el Mandeb strait, and into the Arabian Peninsula [35](see Fig. 1). Ethiopia’s role is further highlighted by its pivotal role as a migration route and hub dating back to the earliest phases of Anatomically Modern Human (AMH) migration [7, 35–37]. Genetics research, involving Ethiopians and Egyptians by Pagani et al. [38] reported that the northern route via Egypt was the exit point for the most recent AMH migration wave (55,000 years ago). The authors of this study argued that the remaining African haplotypes in Egyptians were more similar to non-African populations, and the estimated genetic split time was more recent for Egyptians (55,000 years ago) than for Ethiopians (65,000 years ago), pointing to Egypt as the final dispersal point [38]. However, the Egyptian route was critiqued as inconclusive by Shriner and Keita [39]. Furthermore, the model of a single, human exodus is strongly contradicted by evidences supporting multiple, earlier dispersal waves [40]. The concept often termed the “leaky pipeline” model makes the southern route via Ethiopia (65,000 years ago) the most feasible pathway for large wave of migrations as described by Pagani et al. [38]. Taken together, while Egypt may have facilitated the late migration, archaeological and genetic evidence consistently showed that Ethiopia plays a foundational role in both the “Out of Africa” and “Back to Africa” human migrations and expansion. Ethiopia, as the gateway for the human exodus out of Africa, is supported by its geographical proximity to the southern route and evidence from paleontological and archaeological discoveries. Ethiopia’s proximity to the southern corridor led many to believe it was the primary departure point for early human migrations [7, 35] as illustrated in Fig. 1. Moreover, the earliest AMH fossil evidence in the region, the Omo I remains from the Omo Kibish Formation, was originally dated to approximately 195,000 years ago [43], with a more recent re-evaluation pushing the minimum age to at least 233,000 years ago [1].

Recent genetic studies have revealed that all contemporary African populations carry traces of archaic DNA, but from different sources. East African populations, including Ethiopians, do carry traces of Neanderthal DNA, primarily due to a “back-to-Africa” migration from Eurasia via the Horn of Africa, particularly through Ethiopia [44]. Distinctly, populations in West, Central, and Southern Africa (such as the Yoruba and San) carry archaic DNA that is not Neanderthal, instead derived from an unknown, deeply divergent archaic African hominin that interbred with modern humans within the continent, highlighting a complex history of both Eurasian re-entry and ancient, indigenous African admixture [45].

The presence of Neanderthal genetic components in Ethiopian and other Horn of Africa populations was evident from a report confirming that approximately 0.3% to 1.5% of the genome in admixed ethnic groups like the Amhara constitute Neanderthal’s DNA [46]. The retained genetic components exhibit clear signals of positive selection due to their conferred fitness advantages, especially in enhancing immune system function and adapting to UV radiation exposure [45, 47]. The most notable advantageous variants include archaic haplotypes in the Toll-like Receptor (*TLR*) gene cluster (*TLR1*,* TLR6*, and *TLR10*), which were strongly selected for because they enhanced innate immune responses upon re-exposure to African pathogens [44, 48]. Furthermore, variants affecting skin and barrier function, such as those related to keratin filaments and loci like *Oculocutaneous Albinism Type 2 (OCA2)* and *Basonuclin Zinc Finger Protein 2 (BNC2)*, also show evidence of positive selection, suggesting they offered a crucial adaptive benefit against local environmental stressors and pathogens [46, 49]. This deep history and subsequent genetic diversity within Ethiopian populations shaped by “*Out of Africa*” and “*Back to Africa*” adaptations offer unique insights into human evolutionary history and may inform future research on health and disease across global populations.

## Methodology

To prepare this comprehensive narrative review, we first conducted a systematic search of major electronic databases (PubMed, Scopus, and Google Scholar) specifically aiming to capture literature on the genetic and genomic landscape of the Ethiopian population and context of genomic research in the region. The search strategy was structured into three thematic blocks, connected by “AND,” using truncation (*) to ensure broad retrieval. The full search string used was: (Ethiopia* OR Ethiopian*) AND (genomic* OR genetic* OR ancestry OR admixture OR diversity OR landscape OR population OR selection) AND (precision medicine OR ethical* OR equity OR capacity building OR clinical OR health). The literature selection process involved an initial screening of titles and abstracts, followed by a full-text assessment to determine each article’s contribution to the review’s narrative. To maintain focus and quality, only studies investigating genetic diversity, population structure, or adaptation in Ethiopian populations; discussing human genomics, genetics, or related ethical/policy issues; and explicitly addressing the genetic landscape, evolutionary history, or application/ethics of genomic research within Ethiopia were included. Due to the limited existing literature on human genomic research in Ethiopia, strict exclusion criteria were not applied. Instead, the goal was to synthesize all available information to construct a cohesive argument for readers. However, we excluded non-peer-reviewed abstracts and non-English articles. Following a critical assessment of the retrieved full texts, the findings were thematically organized to not only describe the Ethiopian genomic landscape, but also to build the case for its global significance and a call for inclusive genomic research.

## Genetic signature of adaptive selection in Ethiopian populations

Human populations inhabit diverse environments and face a range of selective pressures, leading to genetic adaptations to local conditions [50–52]. These pressures, such as altitude, sociocultural organization, infections, diet, climate change, and UV exposure, drive natural selection [53–56]. Importantly, the pattern of these genetic adaptations is determined by the timing, strength, and direction of natural selection. On this basis, selection could be positive (adaptive), negative (purifying), or balancing [52]. Positive natural selection promotes the spread of advantageous alleles [50–52, 56], negative selection removes harmful alleles resulting from genetic mutations [52], and balancing selection maintains genetic diversity of a certain traits [57] in a population. Among these, positive selection offers critical insights into human resilience and evolutionary biology, including the interplay between genetics and environment and designing a more inclusive and effective approach in global health and precision medicine.

While early efforts to detect positive selection were hindered by methodological limitations [58, 59], advances in whole-genome scanning have since enabled the identification of genetic loci shaped by natural selection [33, 59, 60]. Genomic researchers have now revealed novel insights into the genetic loci and biochemical pathways shaped by selective pressures [61, 62]. Building on these advancements, this part of the review focuses on (1) detailing genetic markers of positive selection found in the Ethiopian population and (2) describe how adaptation to environmental pressures, such as HA, infectious diseases, or dietary shifts, influenced the genetic diversity of this population. These insights may clarify the advantage of studying Ethiopia’s unique population structure in the context of health outcomes, offering both beneficial and detrimental examples of past adaptations with implications for global populations.

### Positive selection in the Ethiopian population to high altitude (HA) hypoxia

Biological adaptation to hypoxia at HA is one of the most extensively studied examples of positive selection in human populations [15, 18, 34, 61–74]. HA hypoxia puts a lot of physiological strain on human populations, but over time, some groups have evolved mechanisms to tolerate this condition [63, 70]. One such adaptation is the elevation of hemoglobin (Hb) concentration to compensate for reduced oxygen saturation [19]. Another strategy to address HA hypoxia is a metabolic shift that favors glucose oxidation and glycolysis over fatty acid oxidation [75, 76].

Genes in the hypoxia inducible factor (HIF) pathway demonstrate genetic signatures of positive selection, as their variants offer strategic adaptations to HA hypoxia. Researchers identified two key genes, *endothelial PAS domain containing protein 1* (*EPAS1*) and *egl-9 family HIF* (*EGLN1*) related to HA hypoxia [47, 64, 73, 77]. *EPAS1* encodes HIF2α, a transcription factor from the HIF family, and *EGLN1* encodes *Prolyl hydroxylase domain 2 protein (PHD2)*, a prolyl hydroxylase that causes HIF degradation in an oxygen-dependent manner [77]. Genetic variations in the *EPAS1* and *EGLN1* genes, which have been favored by positive selection in HA populations, enable a crucial adaptation, including reduced hemoglobin (Hb) levels. This helps prevent polycythemia, a potentially dangerous condition and supports beneficial physiological responses that enhance survival under chronic hypoxic stress [47, 78]. Another gene, *peroxisome proliferator activated receptor alpha* (*PPARA*), which regulates metabolic responses to hypoxia, has selection signatures in HA Ethiopian [72] and Tibetan populations [75, 79] as detailed in Table 1.

**Table 1:** Candidate genes involved in high-altitude adaptation in Ethiopia

Chromosome region	Candidate Genes	Function in adaptation	References
Ch.10q22.1	*CBARA1*	It is a putative candidate gene that may be involved in the regulation of mitochondrial metabolism by HIF-1, which is considered necessary for the body’s response to hypoxia.	[47]
Ch.1p13. 3	*VAV3*	It is a guanine nucleotide exchange factor (GEF) that regulates Rho family GTPases in vitro and promotes angiogenesis in vivo, a process often driven by HIF-1 under hypoxic conditions. These processes are essential during responses to environmental stress such as hypoxia, inflammation, or injury, allowing tissues to reorganize, heal, and restore function.	
Ch. 15q25.1	*ARNT2*	ARNT2 and THRB are associated with pathways that influence Hb levels and hypoxia responses. ARNT2 may contribute to HIF-mediated signaling in specific tissues, while THRB affects erythropoiesis through thyroid hormone signaling. However, they are not direct sensors of HIF-1 or hypoxia.	
Ch. 3p24.2	*THRB*		
22q13.31	*PPARA*	It regulates the expression of genes involved in fatty acid beta-oxidation and is a major regulator of energy balance.	[34, 47]
Ch. 12p12.1	*BHLHE41*	It plays a dual role in the control of the circadian rhythm and inhibitory transcription factors.	[19, 68]
Ch. 2q36.2	*CUL3*	It plays a crucial role in controlling the ubiquitination and degradation of PHD2 in cells.	
Chr. 11 q13.1	*CORO1B*	It modulates the chemotaxis of human lung endothelial cells and is induced by sphingosine-1-phosphate (S1P).	
Ch. 11q13.2	*ADRBK1*	GRK2-dependent phosphorylation of HuR (Human antigen R) regulates HIF-1α activation under hypoxic conditions.	
19q13	*CIC*	It is a transcriptional suppressor involved in early organ development, and its regulation of cell proliferation and differentiation supports tissue adaptation during development and in response to stress or injury.	[80]
19q13.2	*PAFAH1B3*	It is related with coronary artery disease and organ development. It contributes to cellular adaptation by modulating responses to stress and inflammation.	
19q13.2	*CXCL17*	It promotes angiogenesis and enables tissues to adapt to stressors such as hypoxia, injury, or inflammation.	
19 (q13.1–>13.2)	*LIPE*	It plays a role in hypoxia via lipolysis, triglyceride metabolism, and energy storage.	
	*CNFN*	It is involved in hematopoiesis by supporting the tissue microenvironment and plays a role in adaptation by maintaining epithelial barrier integrity, helping tissues respond to environmental stress.	

HA populations are not genetically uniform. For instance, major high-altitude groups such as those in the Andes, Tibet, and Ethiopia have evolved distinct genetic adaptations to cope with chronic hypoxia, reflecting independent evolutionary responses to similar environmental pressures [63]. While Andean highlanders use the “classic” strategy of elevated Hb, which risks Chronic Mountain Sickness (CMS), a mechanism regulated by genes like *EGLN1* that, when less efficient, promotes a higher red cell mass [81]. Conversely, Tibetans and Sherpas circumvent this danger by possessing specific genetic variants in the *EPAS1* and *EGLN1* loci that actively suppress this Hb response, instead favoring increased ventilation and nitric oxide-mediated blood flow. This in turn results in Tibetan populations maintaining near sea-level Hb concentrations and a significantly lower risk of CMS [36, 72, 82]. Even more distinctively, Ethiopian highlanders especially Amhara, achieve a low-risk, high-fitness phenotype by maintaining both near sea-level Hb concentration and arterial O_2_ saturation through entirely different genetic pathways [19, 83]. Notably, Ethiopians do not share Hb-associated variants found in Tibetans; instead, their adaptation is positively associated with different genes, specifically *thyroid hormone receptor beta* (*THRB*) and *aryl hydrocarbon receptor nuclear translocator 2* (*ARNT2*). These genes were positively associated with Hb levels in Ethiopian Amhara [47], suggesting population-specific adaptations to HA hypoxia.

Alkorta-Aranburu et al. [19], found significant genetic variation among different Ethiopian ethnic groups in their adaptations to HA environments. The HA Oromo population in Bale had Hb levels that were approximately twice as high as the HA Amhara, and they lack genome-wide data for either O_2_ saturation or Hb levels. Conversely, the HA Amhara in the Semien mountains displayed a single significant SNP linked to having lower Hb levels. This contrast is instructive, as the Amhara’s strategy of maintaining lower Hb mirrors the well-known adaptation of Tibetans [83], whereas the Oromo’s high Hb levels suggest a distinct physiological approach to hypoxia. These findings underscore that the variance in physiological markers like Hb saturation may reflect differences in the history, elevation, or duration of HA residence [19]. However, it is essential to note that these conclusions were drawn despite the limitation of small sample sizes typical of such early population-specific genomic studies.

Numerous candidate genes under positive selection involved in HA adaptation were identified by a genomic study that compared the HA Amhara population in northern Ethiopia and low- altitude Omotic ethnic groups in the south, specifically the Aari and Hamer. These genes include *mitochondrial calcium uptake 1 (MICU1)*, which is notably linked to the HIF pathway, and *Vav 3 guanine nucleotide exchange factor (VAV3)* [47]. Another study using SNP genotype data compared five Ethiopian ethnic groups, three that reside at HA (Amhara, Oromo, and Tigrayan) and two that reside at LA (Afar and Agnuak). The study identified only one shared positive selection signal in the *basic-helix loop helix family member e41* (*BHLHE41)* gene, which is part of the HIF-1α response pathway [68]. In addition, positive selection was reported in another group of hypoxia-related genes in Ethiopian populations, including *Cullin3* (*CUL3*), *Beta-adrenergic receptor kinase 1* (*ADRBK1*), *Coronin 1B* (*CORO1B*), and *BHLHE41* (Table 1) [68, 79].

A separate genomic analysis of Amhara and Oromo highlanders identified positive selection signals in genes such as *Lipase E*,* hormone sensitive type (LIPE)*, *Platelet-activating factor acetylhydrolase IB subunit α−1(PAFAH1B3)*,* Cornifelin (CNFN)*, Chemokine (C-X-C motif) ligand 17(*CXCL17)*, and *capicua transcriptional repressor (CIC)*. These genes are involved in lipid metabolism, transcription regulation, and angiogenesis, which are relevant to HA adaptation [80]. The specific functions and locations of these genes are detailed in Table 2. Notably, most of these genes have not been found in prior studies of HA Tibetan or Andean populations [47].

**Table 2:** Candidate genes involved in adaptive selection related to disease resistance, dietary adaptation, sour taste perception, skin pigmentation, and metabolism

Category	Chromosome region	Candidate genes	Function in adaptation	References
Diet related genes	2q21	*LCT*	It encodes the enzyme lactase, which is crucial for digesting lactose, the sugar found in milk and dairy products. Its continued expression into adulthood represents a genetic adaptation to dairy consumption in certain human populations.	[84]
	5q31.2	*TRPP3(PKD2L1)*	It influences how sour tastes are perceived. Strong positive selection has been observed to be acting on two nonsynonymous genetic variants (R278Q and R378W) of this gene in the Ethiopian Gumuz population.	[34, 85]
Metabolism related genes	11q13. 4	*FOLR1& FOLR2*	They encode folic acid receptors that facilitate folate uptake, playing a crucial role in cellular metabolism and supporting adaptation by ensuring adequate folate availability for DNA synthesis and repair, especially during periods of rapid growth or environmental stress.	[34]
	4q23	*ADH*	ADH genes encode enzymes that convert ethanol to acetaldehyde; genetic variations affecting their activity have evolved in some populations as adaptations influencing alcohol metabolism, impacting susceptibility to alcoholism and related diseases.	[86]
	7q21	*CYP3A*	CYP3A encodes the primary isoform of the cytochrome P450 enzyme, which is accountable for the metabolism of exogenous medicines and various other endogenous substrates.	[87]
Skin pigmentation and UV radiation related genes	15q21.1.	*SLC24A5*	SLC24A5 is involved in skin pigmentation and adaptation to varying levels of UV radiation in different environments, helping populations optimize skin protection.	[19]
	11q13.5	*UVRAG*	It promotes DNA double-strand-break repair by directly binding and activating DNA-PK during nonhomologous end joining.	[34]
	9p22.3-p22.2	*BNC2*	This gene codes for a DNA-binding zinc-finger protein that acts as a messenger mRNA-processing enzyme and a transcription factor in skin pigmentation	
	2q21.3	*ZRANB3*	It maintains genomic stability against high UV exposure by facilitating repair and restart of damaged DNA replication forks, preventing mutations and maintaining DNA integrity.	
Immune system related genes	Xq28	G6PD	It encodes an enzyme critical for maintaining redox balance in red blood cells by producing NADPH, which protects cells from oxidative damage. G6PD (mainly G6PDA^−^ variant) deficiency gives protection against severe *P. falciparum* malaria by increasing oxidative stress in infected red blood cells, hindering parasite survival and promoting clearance of damaged cells.	[88–90]
	1q21–25	DARC	It encodes a receptor on red blood cells that *P. vivax* uses to enter the cells; individuals who are Duffy negative lack this receptor on their red blood cells, preventing parasite invasion and thereby providing natural protection against *P. vivax* malaria.	[91–94]
	10p12.33	MRC1	It encodes the mannose receptor, which supports innate and adaptive immunity by recognizing pathogens and clearing cellular debris, thereby aiding adaptation by enhancing immune defense and maintaining tissue homeostasis in changing environments.	[34, 95]
	19q13	IFNL1	IFNL1 encodes a cytokine that is produced in response to viral infection and activates the innate immune response, contributing to adaptation by enhancing antiviral defense at mucosal surfaces and limiting viral spread in diverse environments.	
	9p21.3	INFA family (IFNA14, IFNA16, & IFNA17)	They encode interferon-α proteins that trigger antiviral responses, contributing to adaptation by enhancing the body’s ability to detect and control viral infections across varying environmental exposures.	
	6p21. 3.	HLA	It plays a critical role in the immune system by presenting antigens to T cells. It is also essential for initiating immune responses and for maintaining immune tolerance to prevent autoimmunity. it is often subject to adaptive selection to optimize immune defense across diverse environments.	
	22q13.1	*APOL1*	The G1 and G2 variants of APOL1 became common in some African groups because they provided protection against sleeping sickness an advantage driven by adaptive selection. Despite this benefit, inheriting two copies of either or both of these variants is strongly associated with a much higher risk of developing kidney disease.	[96]
	11p15.4	HbS	It is caused by a mutation in the β globin gene which affects the stability and solubility of the β chain which is inherited autosomal recessive way. This defect is rare in Ethiopia and its association with malaria protection is not well studied.	[97]
	16p13. 3	HbA1 & HbA2	They encodes alpha chains of hemoglobin and mutations in these genes cause alpha thalassemia and it is rarely found in Ethiopians	
Obesity and Type 2 Diabetic susceptibility gene	1p31.1	*NEGR1*	NEGR1, CDKAL1, and TCF7L2 demonstrate a powerful evolutionary conflict: variants that were selected for in the past because they offered a survival advantage (e.g., increased energy efficiency during famine) are the same ones that now contribute to major modern health problems like obesity and Type 2 Diabetes.	[4]
	6p22. 3	*CDKAL1*		
	10q25. 2–q25. 3	*TCF7L2*		

Ethiopians appear to be better acclimated to HA than other groups, showing fewer signs of CMS, especially compared to Andeans [98]. This success is rooted in a distinct physiological strategy that avoids the excessive erythrocytosis seen in Andeans, a key feature of CMS [83]. This difference is reflected at the genetic level; for example, a recent study using whole-genome genotyped data in Papua New Guinea’s highlanders identified candidate genes associated with CMS such as *Serum Amyloid A4*,* Constitutive* (*SAA4)*, *Serum amyloid A1 (SAA1)*,* peroxiredoxin 1(PRDX1)*,* lactate dehydrogenase A* (*LDHA*). The study also implicated the presence of genes from the Notch signaling pathway *presenilin-1 (PSEN1)*,* NUMB endocytic adaptor protein(NUMB)*,* recombination signal binding protein for immunoglobulin kappa J region (RBPJ)*,* Mastermind Like Transcriptional Coactivator 3 (MAML3)* in the population [67]. Yet, none of these genes have been identified in Ethiopian populations, pointing to the existence of a distinct, and possibly more efficient, genetic pathway for HA adaptation [47, 72].

### Human genetic adaptations to local dietary environments

Human metabolism has undergone profound adaptations in response to major dietary shifts throughout history, including the shift from hunter-gatherers to the advent of cooking and the rise of agriculture [99]. These changes imposed strong selective pressures, driving more efficient energy processing and alterations in nutrient utilization. For example, increased amylase gene copy numbers enhanced starch digestion with agrarian diets [100]. Further compelling evidence of positive selection was identified in genes crucial for dairy food adaptation such as lactose persistence (LP) across human populations [100–103]. Such adaptations highlight the dynamic interplay between diet, genetics, and metabolic capabilities, shaping human physiology to accommodate diverse nutritional environments [99]. These findings indicate that human populations have undergone significant genetic changes to better metabolize and utilize the regionally available foods. Such genetic adaptations have conferred survival advantages by improving nutrient extraction or detoxification, leading to the increased prevalence of these beneficial alleles over time.

#### Convergent evolution of lactase persistence (LP) under positive selection

A well-documented example of diet-driven positive selection is LP, the continued activity of the enzyme lactase into adulthood, which enables the digestion of lactose as an energy source [56, 84, 104–107]. While lactase activity typically declines with age, certain populations, including Ethiopians with a long history of dairy consumption exhibit strong genetic signals of positive selection in the *LCT* gene, which encodes the enzyme lactase [84, 106–109]. This selection signature is closely associated with the domestication of animals, long-term adult milk consumption, and shared cultural practices that favored dairy use [108]. The evolutionary advantage of LP likely arose from the additional caloric and nutritional benefits that may have been critical for survival and procreation in periods of food scarcity [110, 111]. Today, nearly 35% of the world’s population exhibits LP into adulthood [112].

In Europeans, LP is primarily linked to the T allele of the SNP-*13910C>T (rs4988235)*, located upstream of the *LCT* enhancer region [113]. This variant’s prevalence across Europe is not uniform but exhibits a geographical gradient [107]. Ancient DNA (aDNA) analysis suggests that the LP-associated allele in Europeans only achieved high frequencies within the last few millennia, indicating that its rapid rise does not correspond directly to a continuous history of dairy consumption stretching back to the original Neolithic transition [114]. Although the European LP variant is the most extensively researched, distinct LP-conferring alleles have been discovered in African and Middle Eastern groups, including *-14010G* and *-13915G* [109]. Outside of Europe, the European-dominant LP allele has been observed in various populations, often linked to admixture [28]. Notably, its presence has also been specifically confirmed in African pastoralist communities [115].

In African and Middle Eastern populations, LP is associated with at least three other distinct allelic variants in the same enhancer region: *-13907G (rs41525747)*,* -13915G (rs41380347)*, and -*14010G>C (rs145946881)* [116, 117]. These variants arose and spread within distinct pastoralist communities that developed independent traditions of dairying. For instance, the East African *LCT* variant *-14010G>C (rs145946881)* occurs at a high frequency (>20%) in the strictly pastoralist Khoe population in South Africa and the Nama of Namibia, strongly suggesting an ancestral connection to East African dairy-consuming populations [28, 118].

A study in the Ethiopian population identified a distinct LP-associated allele, *−14009T>G (ss820486563)* in the *LCT* enhancer region [84] as described in Table 2. The presence of this unique allele highlights an independent evolutionary event for LP within Ethiopia, shaped by the region’s long-standing and distinct history of cattle domestication and traditional dairy practices, which differ from those in other parts of Africa or Europe. These findings illustrate how convergent evolution has shaped population-specific adaptations to dairy consumption, with distinct alleles under positive selection in different regions of the world.

#### Positive selection and copy number variation in the salivary amylase gene

The evolutionary adaptation to high-starch diets is frequently exemplified by the copy number variation (CNV) of the salivary amylase gene, *AMY1*. This is evident from the fact that *AMY1* CNV demonstrated recent positive selection driven by dietary pressure [100, 119]. This gene is crucial for the initial breakdown of starch, and genomic evidence supports *AMY1* CNV as a prime example of human adaptation [119]. Studies globally demonstrate a significant correlation between higher *AMY1* CNV and adaptation to diets rich in starch [100, 119, 120]. Functionally, a higher number of gene copies leads to increased salivary amylase enzyme production, resulting in more efficient starch digestion.

The *AMY1* copy number is not randomly distributed; the Neolithic transition to farming reinforced positive selection for efficient starch digestion, leading to high CNV in agricultural populations compared to fishing, hunting, and pastoral groups [100, 121]. Importantly, this selective pressure is not tied exclusively to farming: the Hadza hunter-gatherers, whose diet relies heavily on starchy underground storage organs, also exhibit high *AMY1* CNV [100]. This pattern underscores that the adaptation is driven by the sustained caloric importance of starch resources, whether wild or cultivated. Individuals with higher *AMY1* CNV gained an evolutionary advantage by being able to extract more energy from carbohydrate-rich diets, improving survival and reproduction, particularly during scarcity. The study of *AMY1* CNV offers crucial insights into gene-diet interactions, but detailed research on this variation is currently lacking in Ethiopia. Given Ethiopia’s deep agricultural history and profound ethnic diversity, it represents an important region for in-depth *AMY1* CNV research.

#### Positive selection in the *TRPP3* gene and the evolution of sour taste perception

The human taste system has evolved in a population-specific manner, adapting to diverse environmental food availability and dietary risks [122]. Among the various taste perceptions, sour taste has been particularly influenced by dietary pressures [123]. Its proposed evolutionary role is to aid in detecting ripe or spoiled, acidic foods, thereby acting as a protective mechanism against consuming harmful substances [124]. Variations among individuals in their ability to perceive and prefer sour tastes are associated with inherited genetic factors [123]. The current understanding of sour taste perception points to *Transient Receptor Potential Polycystin-3 (TRPP3)*, a gene encoding a member of the TRP channel family, primarily expressed in Type III taste cells. *TRPP3* has been identified as a human sour sensing gene due to its high expression in tongue receptor cells and its role in sensing pH changes [125].

Genetic signals of diet-related positive selection at *TRPP3* have been observed in multiple human populations [56]. For instance, a recent study identified adaptive selection driving *TRPP3* loss-of-function in an Ethiopian population, specifically among the Gumuz people [85].This selection signal was detected in two non-synonymous genetic variants of *TRPP3* namely, *R278Q* and *R378W*. *R278Q* inhibits *TRPP3* activation during alkalization, while *R378W* significantly reduces channel activity by altering the voltage sensor domain and slowing channel gating of sour taste buds [34, 85] (see Table 2). Hence, the discovery of *TRPP3* loss-of-function in the Gumuz people of Ethiopia points to a distinct evolutionary path for sour taste perception in this group, likely shaped by their unique diet and environment.

The genetic adaptations to local dietary environments among Ethiopian populations underscore the dynamic interplay between culture, environment, and evolution. The convergent evolution of lactase persistence, variation in amylase gene copy number, and unique adaptations in sour taste perception reflect how dietary practices could leave a lasting imprint on the genome. These examples not only highlight Ethiopia’s rich evolutionary history but also offer valuable insights into population-specific nutritional needs and metabolic health, reinforcing the importance of inclusive genomic research in informing precision nutrition and public health strategies.

### Metabolic adaptations in human populations

The evolution of human metabolism has been shaped by fluctuating food availability and shifting diets throughout prehistory and early civilization [126, 127]. Genes of metabolic function exhibit some of the strongest signatures of positive natural selection in the human genome. This is largely due to evolutionary pressures that favored metabolic adaptations, enabling humans to survive and reproduce in diverse environments [55, 61]. Numerous studies have documented such selection signals, highlighting the importance of human metabolic adaptation to varying ecological and dietary conditions [62, 76, 128, 129].

#### Evidence of positive selection in *CDKAL1*,* NEGR1*,* TCF7L2*, and *PPARA* genes

Compelling evidence suggests that several key genetic loci, namely *Cdk5 regulatory associated protein 1-like 1 (CDKAL1)* [62, 129, 130], *Neuronal Growth Regulator 1 (NEGR1)*, and *transcription factor 7-like 2 (TCF7L2)* [4, 131], along with *PPARA* [76], have undergone positive selection, contributing to human metabolic adaptations. This evolutionary pressure indicates that specific variants within these genes offered a selective advantage, shaping their distinct yet interconnected roles in energy homeostasis and the response to various environmental challenges.

*NEGR1*, for instance, is predominantly expressed in the hypothalamus, a key brain region controlling appetite and energy balance. It is implicated in the regulation of food intake and body weight, with variants even linked to obesity and body mass index, potentially affecting adipocyte lipid trafficking [132, 133]. *CDKAL1* is vital for pancreatic beta-cell insulin secretion and proper protein synthesis through tRNA modification; recent findings also link it to mitochondrial function in metabolically active tissues [129]. *TCF7L2* is a transcription factor integral to the Wnt signaling pathway, significantly influencing pancreatic beta-cell function and insulin secretion, as well as *glucagon-like peptide-1 (GLP-1)* production in the gut, thereby impacting glucose homeostasis [4]. Lastly, the *PPARA* gene encodes Peroxisome Proliferator-Activated Receptor alpha, a crucial transcription factor primarily involved in lipid metabolism. *PPARA* particularly promotes fatty acid oxidation and ketogenesis, especially during times of food scarcity [75, 76, 134]. For example, certain *PPARA* haplotypes have been associated with metabolic adaptations to HA hypoxia in Tibetan [73, 75], Sherpan [76], and Ethiopian Wolaita populations [62].

Signatures of positive selection at *CDKAL1*,* NEGR1*,* TCF7L2*, and *PPARA* genes likely reflect evolutionary pressures exerted by historical fluctuations in nutrient availability during feast-famine cycles (fluctuating food availability) which necessitate to finely regulating energy intake and expenditure in response to this variability. Variants of these genes that promoted efficient energy storage, conserved glucose, or optimized nutrient utilization would have conferred a significant survival and reproductive advantage which implicates signature of positive selection [4, 62, 129, 131, 132, 135]. This phenomenon is in line with the “thrifty gene” hypothesis. According to this hypothesis, ancestral genetic variants that were historically advantageous in enabling individuals to efficiently process food to deposit fat during periods of food abundance. This selection pressure ensured the prevalence of these variants in human populations, as individuals with these “thrifty” genotypes were better equipped to endure scarcity [136]. Variants of these genes were also identified in the Ethiopian population, particularly in the Wolayita ethnic group [4, 62]. A prime example is the *PPARA* gene, demonstrating positive selection due to its critical role in energy metabolism during periods of prolonged food scarcity. This finding aligns with the population’s historical dietary reliance on enset, a high-carbohydrate, low-fat, and low-protein plant domesticated in Ethiopia following probable food deprivation 10,000 years ago and further reflects metabolic adaptation to HA hypoxia [34]. However, these gene variants that conferred an ancestral advantage are now maladaptive in modern food-rich environments, as evidenced by their detrimental associations with conditions like T2DM and obesity [4, 62, 129]. This rapid shift to contemporary lifestyles, characterized by abundant calorie-rich foods and reduced physical activity has thus revealed the adverse health consequences of these formerly beneficial genetic traits.

#### Positive selection in folic acid metabolism genes

Folic acid is the synthetic version of folate (vitamin B9), a nutrient essential for RBC production and is naturally found in various foods [137]. Positive selection in folate metabolism genes is primarily driven by the interplay between dietary folate availability and physiological demand. Key evolutionary pressures include UV exposure and folate intake variability, persistent risk of nutritional deficiencies, and heightened physiological requirements during critical life stages such as pregnancy [34]. These signals of positive selection were observed in diverse Ethiopian ethnic groups like the Gumuz, Wolayita, Somali, Oromo, and Amhara [34, 38].

Genetic variants that enhance folate absorption and metabolism have been shaped by natural selection due to their role in preventing folate deficiency-related conditions like anemia and neural tube defects one of the most studied variants is the methylenetetrahydrofolate reductase (MTHFR) C677T polymorphism, which plays a key role in converting folate into its biologically active form, 5-methyltetrahydrofolate (5-MTHF). The derived T allele is associated with reduced enzyme activity and elevated homocysteine levels, increasing the risk of neural tube defects, particularly in low-folate environments. Despite this, the persistence of the T allele in certain populations may reflect evolutionary pressures or selective advantages that are not yet fully understood [138].

Recent directional positive selection was observed in *folate receptor α−1(FOLR1)* and *folate receptor α−2 (FOLR2)*, members of the folic acid receptor family described in Table 2 in Ethiopian populations, including Amhara, Oromo, Somali, Wolayta, and Gumuz ethnic groups. These receptors are responsible for binding to folic acid and its reduced derivatives, and for delivering *5-methyltetrahydrofolate (5-MTHF)* into cells, a process fundamental to DNA synthesis, cell division, and overall health. The discovery of intense signals of positive selection for these receptors in Ethiopian populations is a profound indicator that a powerful evolutionary dynamic has been at play. This selection pressure was driven by the critical need for enhanced folate uptake and utilization. This heightened requirement was potentially compounded by significant environmental challenges, including specific dietary factors, high UV radiation exposure that degrades folate, or the constant immunological demand imposed by local pathogens [34, 38]. These adaptations likely conferred a survival advantage in environments with low dietary folate or high UV exposure, influencing reproductive success and leaving a lasting mark on the genetic makeup of these populations.

#### Signatures of positive selection in the *ADH* gene family

Studies have revealed positive selection signals in *alcohol dehydrogenase (ADH)* cluster genes, essential for ethanol metabolism by catalyzing its conversion to acetaldehyde [86, 139]. This evolutionary pressure is largely attributed to the widespread consumption of fermented foods and beverages that followed the advent of agriculture, which introduced regular dietary exposure to ethanol [86, 139, 140].

Variants in *ADH* genes, such as the *ADH1B 48 C >T (rs1229984)* mutation, can alter enzyme function and are associated with traits like reduced alcoholism risk. The Ethiopian population specifically shows strong signals of positive selection for this *ADH1B* variant, with evidence suggesting its associated haplotypes were introduced from a Eurasian source within the last approximately 2000 years [86]. Ethiopia has a long history of consuming traditional fermented beverages like *tella*,* tej*,* borde*, and *arake* [141], which are integral to its culture and diet, creating an environment where efficient alcohol metabolism would confer a significant selective advantage. Therefore, the Ethiopian population serves as a compelling case study for understanding how agricultural practices and the resulting dietary shifts have driven recent human adaptation in alcohol metabolism genes.

#### Evolutionary selective signatures in *CYP* genes and drug metabolism

Human cytochrome *P450 (CYP)* enzymes, a diverse superfamily of membrane-bound hemoproteins, play critical roles in detoxifying xenobiotics (foreign compounds like drugs, toxins, and environmental pollutants), as well as metabolizing endogenous substances for cellular homeostasis. They are crucial for detoxifying drugs, processing substances within cells, and maintaining the body’s internal balance. Notably, enzymes from *CYP* families (1–3) are responsible for nearly 80% of oxidative metabolism in humans and contribute to approximately 50% of the overall elimination of commonly prescribed clinical drugs. These genes have been subject to positive selection throughout human evolution as briefly reviewed elsewhere [142]. Variants of *CYP* enzymes that could more efficiently detoxify harmful substances or metabolize novel food components would have conferred a significant survival advantage, leading to their increased frequency in the population [4, 143].

Cytochrome *P450 3 A (CYP3A)* is one of the most important isoforms of the cytochrome *P450* family, playing a critical role in the metabolism of a wide range of drugs and endogenous substrates [144]. Ethiopians display elevated overall *CYP3A* activity and a distinct distribution of *CYP3A5* variant alleles [87], with evidence of positive selection identified within this gene cluster [144]. Strong selective pressure from local diets or toxins favored people with highly active *CYP3A* genes, granting them superior detoxification. This genetic edge improved the body’s ability to neutralize or quickly excrete harmful substances, which led to greater survival and reproduction, thereby making these beneficial *CYP3A* alleles common in the population [142].

Another key member of the cytochrome *P450* family involved in the metabolism of a broad range of therapeutic drugs is cytochrome *P450 1A2 (CYP1A2)*. There is evidence of mild purifying selection operating on *CYP1A2* [4], indicating that mutations impairing its core functions are generally removed from the population. This purifying selection is primarily driven by *CYP1A2*’s essential role in metabolizing both vital endogenous compounds (melatonin and estradiol) and various environmental procarcinogens (polycyclic aromatic hydrocarbons from smokes and heterocyclic amines from cooked meat), acts to remove severely deleterious mutations that would impair its fundamental, conserved functions [145]. Its consistent and accurate function is critical for maintaining cellular homeostasis and preventing the accumulation of harmful substances. Concurrently, positive selection has been detected on specific alleles or regulatory regions of *CYP1A2* in various populations [124]. This positive selection likely reflects adaptations that fine-tune its activity in response to localized dietary components like varying levels of caffeine consumption or exposure to specific plant-derived compounds or unique arrays of environmental pollutants encountered throughout human evolutionary history. Notably, *CYP1A2* is preferentially expressed in the liver, accounting for a substantial 13–15% of the total hepatic *CYP* content [124, 146–148], highlighting its significant contribution to overall drug and xenobiotic metabolism compared to many other individual *CYP* enzymes as described in (Table 2). Genetic variations in *CYP1A2* are notably associated with adverse reactions and altered therapeutic efficacy for specific classes of medications, including antipsychotics like clozapine, olanzapine and certain antidepressants such as fluvoxamine, where altered metabolic rates can lead to toxicity or therapeutic failure [142]. This is strikingly illustrated by a detailed analysis of the *CYP1A2* gene across five Ethiopian ethnic groups (Afar, Amhara, Anuak, Maale, and Oromo) which revealed an exceptionally high level of overall genetic diversity, including numerous novel variants previously unrecorded in global databases. Although phylogenetic analyses showed relative tight clustering among these specific Ethiopian ethnic groups, their collective gene diversity was nearly double that of all other global populations combined [4].

The need for population-specific data extends beyond drug-metabolizing enzymes to drug target genes. For instance, the genetic markers of warfarin dose response, predominantly including markers of warfarin sensitivity caused by common polymorphisms in the major warfarin target gene *VKORC1* and its metabolizing enzyme *CYP2C9* [149]. *VKORC1 Asp36Tyr* polymorphism, a variant associated with a high requirement for the anticoagulant warfarin (i.e., warfarin resistance), has been found to be common in individuals of Ethiopian descent. Specifically, carriers of this variant required significantly higher warfarin doses compared to non-carriers [150]. Another important highlight is the case report published in the New England Journal of Medicine that highlights a specific instance of codeine intoxication in an Ethiopian patient linked to ultrarapid CYP2D6 metabolism [151]. These finding, alongside the heterogeneous *CYP* profiles [4, 152], confirms that current pharmacogenomic panels primarily based on variants common in European or East Asian populations are insufficient for accurately predicting safe and effective drug dosing for Ethiopian individuals.

### Human adaptive evolution to ultraviolet radiation

Human skin pigmentation is a classic example of natural selection, shaped by the evolutionary necessity to protect our skin from harmful UV radiation. This process has fine-tuned melanin levels in our skin to correspond to regional UV intensity, resulting in two broad, gradual geographical gradients of skin color. The evolution of skin pigmentation diversity through such adaptive evolution in the human population has been comprehensively reviewed [153]. Given Ethiopia’s geographical position, which exposes its populations to generally high UV index levels, and its rich genetic and environmental heterogeneity, this region provides a unique context to explore local adaptations in genes related to skin pigmentation, potentially identifying novel or refining known adaptive pathways.

Genetic analyses of various African populations have identified specific variants in or near several genes like *Solute Carrier Family 24 Member 5(SLC24A5)*, *major facilitator superfamily domain containing 12 (MFSD12)*, *Damaged DNA binding protein 1 (DDB1)*, *Transmembrane protein 138 (TMEM138)*,* Oculocutaneous albinism type 2(OCA2)*, and *HECT domain AND RCC1-LIKE dmain 2(HERC2)* that are strongly associated to skin pigmentation [154]. In East African populations, lighter skin pigmentation has been strongly associated with variants in the *SLC24A5* gene, which are thought to have originated through gene flow with non-African populations [155]. Notably, positive selection on *SLC24A5* has been observed in both European and Ethiopian populations [156]. The locus of this gene demonstrated particularly strong selection signals in the Wolayita ethnic group. Specifically, the alleles of SNPs rs1426654 and rs1834640 within the *SLC24A5* gene, which are associated with light skin pigmentation in Eurasian populations, were found at a high frequency (47.9%) in this indigenous Ethiopian [62]. While the exact evolutionary pressure driving this selection in Ethiopia post-admixture is complex and a subject of ongoing research, the prominence of these light-skin-associated variants points to a unique adaptive history influenced by gene flow.

Beyond *SLC24A5*, positive evolutionary selection signals have also been identified in the *UV Radiation Resistance Associated Gene (UVRAG)* locus, linked to protection against UV-induced damage and the modulation of skin pigmentation [153]. In Ethiopian populations, a specific locus within *UVRAG* has been detected under positive selection across multiple ethnic groups, including the Somali, Amhara, Oromo, and Wolayita. Notably, the strongest positive selection signals for *UVRAG* were observed in Cushitic group (Oromo and Somali) populations [34], suggesting a particularly significant adaptive advantage in these groups, potentially reflecting intense or prolonged exposure to high UV environments. This strong selection signature is functionally coherent, as *UVRAG* is known to act as a tumor suppressor [157] and, critically for adaptation to UV radiation, activates the nucleotide excision repair pathway, which directly protects cells from UV-induced DNA damage [158]. Therefore, the selection on *UVRAG* likely represents a key genetic adaptation enhancing cellular resilience against high UV exposure in these Ethiopian populations.

A study reported that the Wolayita and Gumuz ethnic groups share a specific variant within the *BNC2* gene, which has been implicated as a marker of positive selection for skin pigmentation. This variant is particularly notable because it contains a potential regulatory shift in an intron region of the gene, likely impacting *BNC2* expression by altering regulatory elements that control its transcription or processing [34]. The *BNC2* gene encodes a zinc-finger protein that functions as a transcription factor and *mRNA* processor, binding to DNA [159]. It is expressed in both keratinocytes and melanocytes, and crucially, higher expression levels of *BNC2* have been linked to darker skin pigmentation [160]. The prevalence and positive selection of this particular *BNC2* variant in the Wolayita and Gumuz populations strongly suggest it drives increased *BNC2* expression, thereby contributing to darker skin pigmentation. This adaptive response is highly relevant for populations exposed to high levels of UV radiation, common in their ancestral East African environments, providing enhanced protection from UV radiation.

The finding regarding the *BNC2* gene in the Wolayita population appears to contrast with the high frequency of the light-skin-associated *SLC24A5* variant found in the same group. Specifically, the derived *SLC24A5* allele associated with lighter pigmentation in Eurasian populations is present at a high frequency in Wolayita people [62]. The origin of this *SLC24A5* variant in the Wolayita population is debated; while its presence is often linked to gene flow from non-African populations such as the Levant [61, 62], analysis of ancestral versus derived allele frequencies in this specific group suggests a less clear or potentially different genetic legacy. This apparent contradiction underscores the complex, polygenic nature of skin pigmentation and suggests that a single population can be influenced by multiple, and at times conflicting, evolutionary pressures and genetic legacies [61, 62]. For example, while the high-frequency *SLC24A5* allele may reflect ancient or recent gene flow into the region, intense local UV radiation could have simultaneously driven selection for other traits, such as those linked to *BNC2* that enhance darker pigmentation or improve UV protection.

Furthermore, the *Zinc Finger RANBP2-Type Containing 3 (ZRANB3)* gene has garnered attention due to its substantial correlation with skin pigmentation and evidence of positive selection [34]. *ZRANB3* plays a critical role in maintaining genomic stability, particularly during DNA replication, where its key function is to resolve replication stress [161]. This function is highly pertinent to UV exposure, as the absence of *ZRANB3* can lead to genomic instability and hypersensitivity to various DNA-damaging stimuli, including UV radiation itself [34]. Therefore, the observed positive selection signals for *ZRANB3* in this population, likely reflect an adaptive response to high UV environments, where maintaining DNA integrity in the face of constant UV-induced damage confers a significant selective advantage.

### Human genetic adaptations to infectious diseases

Throughout human history, populations have been engaged in a continuous evolutionary arms race with pathogens, making genetic adaptation a fundamental requirement for survival. This persistent interaction has exerted strong selective pressure on the human genome, shaping its structure and function over time. Pathogens, through their continuous challenge to host defenses, have driven the propagation of alleles that enhance resistance or modulate immune responses, thereby influencing patterns of genetic variation across populations [52, 101].

Recent genomic studies continue to uncover widespread evidence of positive selection in the human genome in response to selective pressure exerted by infectious diseases [162]. For instance, human genes interacting with pathogens like *Yersinia pestis* (plague), HIV-1, and Ebola virus demonstrate strong signatures of positive selection across diverse populations, reflecting ancient and ongoing exposure to these threats [52, 163]. Furthermore, recent analyses of human genetic adaptation to SARS-CoV-2 infection have identified variants in host genes, like *Angiotensin-converting enzyme 2 (ACE2)* and *Transmembrane serine protease 2(TMPRSS2)*, that appear to be targets of selection, possibly driven by prior viral infections [164]. Genomic signatures of selection have been identified in an Amerindian population, specifically adapted to combat a relatively high pathogen burden dominated by helminth infections, showcasing how pathogens directly shape host genetic variation [165]. Importantly, infectious diseases such as malaria are one of the most powerful selective forces that induce positive selection in the human population [52]. Positive selection of numerous protective alleles, particularly within populations exposed to endemic forms of the disease. Classic examples include *glucose-6-phosphate dehydrogenase (G6PD)* deficiency [166], variants in the Duffy blood group that provide resistance to *Plasmodium vivax* [167], and the sickle cell trait (*HbS*) [168]. These insights highlight how infectious diseases remain a powerful driving force in human evolution, with implications for understanding population-specific disease susceptibility and informing public health strategies.

#### Positive selection in *G6PD* gene

A systematic review and meta-analysis have confirmed that positive selection on genetic variants within the *G6PD* gene, particularly the *G6PD* A^−^ deficiency allele which is prevalent in Africa, has driven immune adaptations that confer significant resistance to malaria in both hemizygous males and heterozygous females [91, 168]. The selective force driving this positive selection is the protection offered against severe malaria, as individuals with these variants have RBCs that are less hospitable to *Plasmodium falciparum (P.falciparum)* parasites. However, this protective mechanism comes with health implications, as *G6PD* deficiency can lead to hemolytic anemia when individuals are exposed to certain drugs (like anti-malarial or some antibiotics) or fava beans [169]. Despite multiple reports indicating a general prevalence of G6PD deficiency in Ethiopia [88, 169], the commonly observed G6PD*A− (G202A) and Mediterranean (C563T) variants were not detected in Ethiopian samples [88, 170].Its absence suggests that alternative, yet-to-be-characterized G6PD variants or distinct adaptive mechanisms may underlie malaria resistance in this population.

#### Positive selection in *DARC* gene

The *Duffy Antigen Receptor for Chemokines (DARC)* gene is an example of positive selection driven by infectious disease pressure, particularly by *P.vivax* malaria. *DARC* encodes a chemokine receptor expressed on the surface of RBCs, which *P. vivax* utilizes as a crucial entry point for infecting RBCs [167]. A key variant, the Duffy-null allele (FY*O or rs2814778), arises from a mutation in the gene’s promoter region that diminishes the expression of the *DARC* protein on RBCs. This prevents *P. vivax*’s Duffy binding protein (*PvDBP*) from attaching to RBCs, thereby blocking parasite entry [52, 171]. As a result, individuals who are homozygous for this null allele are consequently highly resistant to *P. vivax* infection. The increased frequency of individuals lacking *DARC* or carrying this mutation within malaria-endemic regions highlights strong positive selection acting on this gene [91–94, 172, 173]. Due to this significant protective advantage, the Duffy-null allele has been driven to near fixation in many sub-Saharan African populations, making it one of the most geographically differentiated selection signatures in the human genome [174]. However, recent studies, including those in Ethiopia, have demonstrated the emergence of *P. vivax* infections in Duffy-negative individuals [91–94, 172].

#### Positive selection in the *Hemoglobin’s globin (Hb)* genes

Hb, the protein responsible for oxygen transport in RBCs, is directly targeted by *P. falciparum* parasites during their life cycle. However, genetic mutations in genes encoding Hb’s alpha and beta globin chains play a central and well-documented role in human adaptation to malaria. These specific mutations alter Hb’s structure or production, resulting in various genetic conditions collectively termed hemoglobinopathies. This creates an environment within the RBCs that is less hospitable for parasite growth, survival, or replication, thus conferring a protective advantage against malaria [175, 176]. These adaptations primarily operate through non-immunological mechanisms, altering the RBC’s susceptibility to infection or promoting earlier clearance of infected cells.

For instance, the *beta-globin gene (HBB)*, located on chromosome 11, provides the genetic blueprint for one of the two main protein components of adult Hb. A specific single point mutation within *HBB* alters this blueprint, resulting in the production of an abnormal Hb variant; *HbS.* Having one copy of *HbS* of the sickle cell gene where at least one of the two beta-globin genes carries a single mutation may help shield carriers of this gene against malaria. However, having two copies *(HbSS)* may increase the risk of dying from sickle cell disease [177, 178]. Mechanistically, this protection is multifaceted: RBCs containing *HbS* tend to sickle under low oxygen tension conditions, such as those found in the capillaries where parasites proliferate. Sickling can impede parasite growth and lead to the premature destruction and splenic clearance of infected RBCs [175].

Another example of genetic adaption to malaria is α-thalassaemia, a condition which occurs when one or more of the four alpha-globin genes that make up part of the Hb molecule are missing or damaged. It has been shown that α-thalassaemia also confers protection from malaria in in both heterozygotes and homozygotes, though the level of protection may not be as pronounced as with sickle cell trait [179]. The protective mechanisms of α-thalassemia, stemming from reduced alpha-globin synthesis that results in microcytosis (formation of RBCs that are smaller than normal), are multifaceted and directly contribute to malaria resistance. These include an increased RBC count, which buffers against the severe anemia characteristic of malaria, and altered RBC properties that both reduce *P.falciparum* parasite growth within the cell and enhance splenic clearance of infected RBCs [176]. Notably, a relatively low prevalence of sickle cell disease (SCD) and α-thalassemia was reported in Ethiopia compared with populations of Western and Central Africa, likely reflecting regional differences and ecological patterns of *P. falciparum* malaria transmission [97, 180, 181].

#### Positive selection in the *APOL1* gene

The *APOL1* gene encodes Apolipoprotein L1, a circulating protein that is a component of high-density lipoprotein (HDL). Its primary physiological function is as an innate immune effector that provides protection against certain species of Trypanosomes which cause Trypanosomiasis (African sleeping sickness) [182]. Genetic variations in the *APOL1* gene are a prime example of adaptive selection driven by infectious disease. While two copies of *APOL1* risk alleles ((G1/G1, G2/G2, or G1/G2)) protect against specific strains of the parasite, they concurrently increase susceptibility to end-stage renal disease (ESRD) [96, 183–185]. This selection signal is driven by the G1 and G2 variants’ ability to bypass the parasite’s defense mechanism, thereby enhancing the host’s innate immune response against the parasite [186]. These protective yet risky alleles are highly prevalent in West African countries, directly correlating with the historical endemicity of the parasites in the region [187]. However, the incomplete penetrance of the *APOL1* high-risk genotype, reflected by the 15–20% estimated lifetime risk of ESRD, confirms its role as a necessary but insufficient genetic susceptibility risk factor. As result *APOL1* risk alleles are considered a probabilistic factor, not a deterministic one [188]. In contrast, the G1 and G2 variants are found at significantly lower frequencies in East African populations, including Ethiopia. This lower prevalence in Ethiopia, particularly in the tsetse fly-absent highlands, where *Trypanosoma brucei rhodesiense* is consequently rare, results in a reduced incidence of ESRD, including HIV-associated nephropathy, in the Ethiopian population compared to individuals of European or West African descent [189].

#### Positive selection in *MRC1* gene

The *MRC1* gene encodes the Mannose Receptor C-type 1q, also known as *CD206*, belonging to the C-type lectin superfamily and plays a critical role in antigen presentation and the clearance of endogenous compounds. This protein is a crucial pattern recognition receptor within the innate immune system, primarily expressed on the surface of macrophages and dendritic cells. Its fundamental immune function involves binding to specific high-mannose carbohydrate structures found on the surfaces of a wide range of pathogens, including various bacteria, viruses, and fungi. This binding initiates the phagocytosis of these pathogens for their clearance and subsequent antigen presentation to T cells, thereby bridging the innate and adaptive immune responses [95].

Genome sequencing analysis has discovered that the *MRC1* gene is another important immune-related strong signal of positive selection in Ethiopian populations, driven by the necessity for enhanced immune defense against the diverse array of pathogens historically prevalent in the region [34]. The specific *MRC1* variants under selection likely optimize the receptor’s function, leading to more efficient pathogen recognition, uptake, and improved downstream immune responses, providing a crucial evolutionary advantage against endemic infections. This receptor is expressed in macrophages, dendritic cells, and nonvascular epithelium where it binds to a wide range ligands [190]. Antigens internalization by *MRC1* can lead to processing for cross-presentation on antigen-presenting cells, which in part hinders the capacity of T cell cytotoxic functions, thereby resulting in immune tolerance to particular antigens [191]. Studies link *MRC1* variants to susceptibility or resistance in various diseases, including inflammatory conditions like asthma and sarcoidosis [192, 193], and critically, infectious diseases such as leprosy [194] and tuberculosis [195]. This highlights the direct benefit these positively selected variants confer to the host’s ability to combat infectious threats, reflecting an evolutionary adaptation to the local pathogenic environment.

#### Positive selection in *HLA* genes

The *Human Leukocyte Antigen (HLA)* genes, residing within the Major Histocompatibility Complex (MHC), represent a paramount example of positive selection driven by the ceaseless co-evolutionary struggle with pathogens. These highly polymorphic genes encode cell-surface proteins essential for binding and presenting pathogen-derived peptides to T lymphocytes, thereby initiating robust adaptive immune responses [196, 197]. The extraordinary allelic diversity observed in *HLA* genes across human populations is a direct consequence of this intense selective pressure, as hosts continually adapt to a vast and evolving array of infectious agents.

Pathogens directly drive the remarkable diversity of *HLA* by constantly evolving mechanisms to evade immune recognition. In response, positive selection primarily operates through balancing selection. This involves negative frequency-dependent selection, where rare *HLA* alleles gain a selective advantage against pathogens that are less adapted to circumvent immune responses mediated by them. Concurrently, heterozygote advantage ensures that individuals carrying two different *HLA* alleles at a given locus can present a wider array of pathogen peptides to T cells, thus conferring a broader spectrum of resistance to diverse infectious agents [197]. These mechanisms collectively ensure the maintenance of extensive *HLA* polymorphism within populations, providing a flexible immune repertoire to combat continuously evolving threats. Compelling evidence of this adaptive process is particularly evident in East African populations, including those from Ethiopia, reflecting a long history of intense pathogen exposure [198, 199]. Recent genomic analyses in diverse Ethiopian ethnic groups have revealed distinct patterns of *HLA* allele frequencies and haplotype structures that deviate from neutral evolutionary expectations. These unique selective signatures indicate adaptive responses to local infectious disease burdens from endemic pathogens such as *P. falciparum* malaria, tuberculosis, and HIV [34, 62]. Such investigations consistently highlight the high allelic diversity within these populations, underscoring the vital role of *HLA* in shaping the immunological resilience of East African populations in response to their specific pathogen landscape.

#### Positive selection in *INF* gene family

The interferon (*IFN)* gene family (*IFN-α14*,* IFN-α16*, and *IFN-α17*), encodes crucial proteins that serve as the body’s first line of defense against pathogens. These proteins are a cornerstone of the innate immune system, playing an essential role in mediating potent anti-viral, anti-cancer, and anti-proliferative immune responses by signaling to other cells to induce an antiviral state, inhibit viral replication, and modulate immune cell activity [200].

Previous study suggested that population-specific positive selection has shaped the immune response in two Ethiopian ethnic groups. In the Amhara population, selection acted on members of the Interferon-alpha (IFN-α) gene family, likely favoring subtle coding variants that enhance the host’s ability to combat a historical high burden of viral infections. Conversely, a distinct signal of positive selection was identified in the Wolayita population in the regulatory regions of the Interferon-Lambda (IFN-λ) genes, specifically in the upstream and intergenic positions between IFN-λ1 and IFN-λ2. Since IFN-λ proteins (Type III interferons) serve as a critical defense at mucosal surfaces, this selection was likely driven by endemic viral pressures with mucosal tropism. The positioning of the signal suggests that regulatory variants were favored, leading to altered expression levels or timing of IFN-λ production specifically, a quicker or stronger induction to provide a more effective early antiviral response and confer a survival advantage [34].

## Genetic signals of positive selection and their health implications

Understanding the health and disease implications of positive selection in Ethiopian populations is crucial for advancing public health, particularly in the context of precision medicine. Ethiopia’s remarkable genetic diversity, shaped by unique evolutionary pressures, underscores the limitations of a generalized approach to healthcare [5, 34, 62]. This rich genetic landscape provides a powerful framework for developing tailored medical interventions that move beyond broad recommendations towards more precise, individualized care.

Adaptive selection in genes involved in human adaptations to local dietary environments may impact human health positively or negatively. The typical examples are adaptive selection in *LCT* and *AMY1* genes. As discussed earlier, *LCT* gene and its variants are responsible for LP in many Ethiopian groups, enabling milk digestion into adulthood which enable them to use milk sugar during periods of shortage of other carbohydrate rich foods [16, 84, 106]. This adaptation highlights how genetic predispositions can influence nutritional strategies. On the other hand, adaptive selection in *AMY1* gene conferred a fitness advantage by optimizing nutrient extraction from starch-rich food sources. However, the health implications in modern contexts are complex; while some studies suggest a role in glucose homeostasis, others show conflicting associations with obesity risk, potentially influenced by gene-diet interactions [201, 202]. Given the diverse dietary patterns prevalent across Ethiopian populations, investigating variants of these genes is critical to provide crucial insights for developing personalized nutrition and effective public health strategies tailored to this unique demography.

Human metabolism has undergone genetic adaptations in response to fluctuating nutrient availability, driven by evolutionary pressures. While these adaptations were historically vital for survival, they now frequently contribute to chronic metabolic dysregulation, significantly impacting health in contemporary populations. For example, in Ethiopians and other human groups, genes like *CDKAL1*,* NEGR1*,* TCF7L2*, and *PPARA* are linked to an elevated risk of metabolic dysfunction, including T2DM and obesity [4, 62, 129, 131, 132, 135]. Genetic variants in these genes are associated with these conditions with varying effect sizes and confidence intervals. The *TCF7L2* gene, particularly the rs7903146 risk allele, represents a significant genetic risk factor for T2DM, with studies reporting odds ratios ranging from 1.35 to 1.74 [203]. Likewise, the rs10946398 polymorphism in the *CDKAL1* gene is associated with an elevated risk of T2DM, as a meta-analysis determined an odds ratio of 1.17 (95% CI: 1.07–1.28) [204]. The *NEGR1* gene is closely linked to obesity, and in one population-specific study, the rs2815752 variant showed a substantial odds ratio of 3.03 (95% CI: 1.19–7.72) [205]. Lastly, variants in the *PPARA* gene, such as the intron 1 C-allele, have been shown to influence the age of onset of T2DM [206]. Understanding the evolutionary underpinnings of metabolic risk is crucial for public health, regardless of the precise genetic mechanism.

The ‘Thrifty Gene’ hypothesis offers an evolutionary framework to explain the increase in susceptibility of populations to metabolic disorders such as obesity and type 2 diabetes mellitus (T2DM). However, recent genome-wide association studies could not identify strong evidence supporting this hypothesis [207, 208], highlighting instead the complex role of gene–environment interactions, potentially shaped by natural selection or genetic drift [209]. Despite these limitations, the hypothesis remains valuable for two key reasons: first, it provides an evolutionary context for understanding population-level vulnerability to metabolic diseases [137]; and second, it underscores the importance of developing personalized prevention strategies that account for the interplay between ancient genetic architecture and modern lifestyles in addressing the global rise of metabolic disorders.

Another critical example of adaptive selection with significant health implications is the widespread prevalence of the Duffy-null allele in many African populations including Ethiopians living in malaria endemic areas. Historically, this genetic variant was understood to provide significant genetic protection against *P*,* vivax* malaria [171]. However, recent studies, including those in Ethiopia, have demonstrated the emergence of *P. vivax* infections in Duffy-negative individuals [91–94, 172]. This phenomenon indicates that the Duffy-null allele no longer confers absolute protection, necessitating re-evaluation of public health screening and intervention strategies.

The genetic variants that cause *G6PD* deficiency due to positive selective pressure against malaria is also a crucial marker in pharmacogenomics and personalized medicine. These variants offer a survival advantage by making RBCs less hospitable to the malaria parasite. However, they make individuals with the deficiency vulnerable to oxidative stress and put them at risk for severe drug-induced hemolytic anemia. For example, drugs like the anti-malarial primaquine and the antibiotic nitrofurantoin can trigger the destruction of RBCs in *G6PD*-deficient individuals. To prevent these dangerous adverse reactions, clinicians use genetic testing to screen for the *G6PD* genotype before prescribing these medications. This allows them to choose safer alternative drugs or adjust dosages, creating a more tailored and effective treatment plan that is directly informed by the patient’s genetic makeup [210, 211].

The *HLA* gene polymorphism, a direct consequence of positive selection driven by pathogen-host co-evolution, profoundly shapes immune function and health outcomes in Ethiopian populations [62, 212]. This extensive genetic diversity, while crucial for mounting broad immune responses, simultaneously influences susceptibility to various diseases, pharmacogenomic profiles, and transplantation compatibility. Recent genomic researches in the Wolayita ethnic group have identified novel signals of positive selection within several *HLA* loci, notably overlapping with regions previously associated with podoconiosis, a prevalent geochemical lymphedema, reinforcing its likely immune-mediated etiology [62, 213, 214]. Further evidence for this immune involvement comes from a similar study highlighting immune activation in podoconiosis pathogenesis [215]. Moreover, a study in North-West Ethiopia linked HLA-A*03:01 variant with an increased risk of Visceral Leishmaniasis progression in HIV-positive individuals [216]. Such findings demonstrate that *HLA* screening can be leveraged beyond routine transplantation and drug safety to genetically stratify risk for both communicable and non-communicable immune-mediated diseases, enabling the transition from generalized public health measures to highly targeted, preventive interventions and tailored monitoring protocols based on an individual’s unique genetic code.

The health implications of *HLA* adaptation also extend to autoimmune conditions and personalized medicine. For instance, a recent study identified high *HLA* risk haplotype (*HLA-DQ2/DQ8* status) frequency for celiac disease (one of autoimmune diseases) in Ethiopian children. This confirms the direct clinical link between *HLA* adaptation and this disease. So, in the context of celiac disease, personalized medicine is primarily about using an individual’s *HLA-DQ2/DQ8* status to tailor screening, diagnosis, and long-term risk management of the condition [217]. Therefore, understanding these population-specific *HLA* adaptive landscapes is therefore fundamental for advancing precision medicine, enabling tailored interventions that account for Ethiopia’s unique genetic heritage.

A remarkably low prevalence of high-risk *APOL1* variants for kidney disease were observed in Ethiopian population [189]. This is in contrast with populations of West Africans, where *APOL1* variants are a major genetic contributors to ESRD and HIV-associated nephropathy [96, 183–185]. However, the assertion that the *APOL1* high-risk genotype exhibits incomplete penetrance and requires other factors such as such as inflammation or viral exposure for the development of ESKD [218]. Reduced frequency of risk variants of *APOL1* gene in Ethiopians means that clinical efforts must instead prioritize other local risk factors of ESRD.

Other critical genetic factor influencing pharmacogenomics and personalized medicine is the genetic polymorphisms in the *CYP450* enzyme superfamily. These genetic variations can alter enzyme activity, leading to different drug metabolism rates among individuals. For instance, some people are slow metabolizers of the *CYP2D6* enzyme, which can cause drug buildup and side effects, while others are fast metabolizers who clear drugs too quickly for standard doses to be effective [4, 152]. Evolutionary pressures have also shaped other drug-metabolizing genes, such as the unique distribution of *CYP3A5* variants [4] and the substantial variation in *CYP1A2* found in Ethiopian ethnic groups. This variability extends to Warfarin-response genes (*CYP2C9* and *VKORC1*), a concern recognized by the U.S. FDA, which has noted that dosing algorithms developed primarily on European and Asian populations may require adjustment for individuals of East African/Ethiopian ancestry [219]. This highlights the necessity for localized pharmacogenomic research to tailor drug therapies [4, 220] and implement personalized precision medicine.

To summarize, the health implications of positively selected genetic variants in Ethiopian populations are profound and multifaceted. While some adaptations confer resilience against environmental stressors and infectious diseases, others may predispose individuals to chronic conditions in modern contexts. Recognizing these dual outcomes is essential for developing personalized healthcare strategies that are both culturally and genetically informed. This underscores the importance of integrating Ethiopian genomic data into global precision medicine initiatives.

## Capacity building for implementing precision medicine in Ethiopia: opportunities and challenges

Despite growing awareness, African populations particularly those in Ethiopia, with their deep evolutionary history and unique genetic diversity remain significantly underrepresented in global genomic research [31]. This critical gap has tangible consequences: it constrains the portability of genetic findings to African settings, results in poor predictive performance of genetic risk scores, and ultimately worsens health disparities. These limitations hinder global efforts in translational research and represent a missed opportunity for precision medicine [31, 221]. While precision medicine has seen success in the Western world, its implementation in many African nations faces significant hurdles. The challenges stem from a lack of comprehensive data, insufficient Pan-African collaboration, and a shortage of skilled healthcare professionals [31, 222].Therefore, advancing genomic research in Ethiopia and the wider African continent is not just a scientific endeavor but a crucial step towards building a truly inclusive genomic ecosystem to implement precision medicine.

Advancement of genomic research in Africa countries like Ethiopia is hindered by a confluence of systemic, ethical, legal, infrastructural barriers, and inadequacy of human genomic research literacy [223]. The primary administrative hurdle is the inefficient ethical review process, which is a common bottleneck in Africa that delays critical project initiation and discourages essential international collaborations. This inefficiency is compounded by the absence of a legal framework to govern the acquisition, storage, and sharing of genomic data is a significant gap, posing risks and undermining trust in the research process [224–227]. On top of these, inadequacy of human genomic research literacy among all key stakeholders, from researchers to participants and the Research Ethics Committees themselves also hindered genomic research in the country as a qualitative research study exploring the perspectives of Research Ethics Committee members in Ethiopia highlighted. This lack of knowledge contributes to unsatisfactory consent and a lack of awareness regarding the vulnerability of human genomic research participants [226]. Another set of challenges are infrastructural. Ethiopia lacks foundational research infrastructure, which limits the scope and scale of genomic studies. Specifically, there is an absence of requisite laboratory and bioinformatics capacity, which are essential for modern genomic analysis [228, 229]. The global genomics market was valued at USD 32.65 billion in 2023 and is projected to reach USD 94.86 billion by 2030 [230].

To overcome these challenges, there is a critical need for large-scale, inclusive genomic studies across Ethiopia. This effort must be supported by sustained investment in training a new generation of Ethiopian researchers and building robust infrastructure, including advanced sequencing facilities, biobanks, and secure data repositories. A vital strategy involves collaborating with senior Ethiopian geneticists in the diaspora to transfer knowledge and skills via training of Ethiopian postgraduate students in Ethiopian universities working in this area. The senior author, Dr. Mersha is a strong advocate for reimagining how healthcare can benefit to everyone in the era of precision medicine, specifically addressing gaps in genomic research through the inclusion of populations that are representative of the global community. As part of his global outreach, he co-founded the Alliance for Research, Innovation, and Education [231], an international team of volunteer educators, researchers, and innovators. Through AfRIE, he initiated, led, and established the first Public Health Genomics (PHG) Graduate Program at Bahir Dar University, Ethiopia. These efforts have not only created infrastructure and a genomics workforce but also highlighted the importance of including representative populations in genomic studies to achieve universal utility in genomic medicine. Such strategic partnerships between Ethiopian institutions and international researchers are essential to foster knowledge transfer and create a self-sustaining genomic discovery ecosystem. These initiatives will not only significantly improve healthcare outcomes in Ethiopia through tailored diagnostics and treatments but enrich global genomic databases with data from one of the world’s most genetically diverse regions. Building a sustainable genomic research ecosystem in Ethiopia will not only improve local healthcare outcomes but also contribute valuable insights to the global scientific community.

## Limitations of the review

While this review provides a comprehensive overview of Ethiopia’s genomic landscape, the scope and conclusiveness of this review are inherently constrained by the current state of published genomic literature on Ethiopia and the broader East African region. Much of the existing research has primarily focused on population diversity and human adaptive traits such as HA tolerance, gene flow, admixture, dietary adaptations, mitochondrial DNA (mtDNA) and ancient DNA (aDNA). Recently, efforts have been made to establish the Ethiopia Genome Project, which includes two phases: a low-coverage dataset of 225 Ethiopian and Egyptian genomes aimed at tracing human migration routes out of Africa (registered in 2010) [232], and a high-coverage dataset cataloging variants from 300 individuals across six Ethiopian populations for demographic history and diversity (released in 2015) [232]. Studies on HA adaptation have sequenced whole genomes of Amhara and Oromo individuals to identify genetic mechanisms underlying survival in hypoxic environments [233]. The Mota ancient genome project produced the first ancient African genome from a 4,500-year-old male, shedding light on Eurasian admixture into the Horn of Africa [234]. Additional work includes mitochondrial DNA heritage studies exploring maternal lineages between Ethiopia and Yemen, conducted using 270 Ethiopian and 115 Yemeni mtDNA sequences [235], and genome-wide SNP panels covering over 60 Ethiopian ethnolinguistic groups to analyze population structure and admixture patterns [236]. While these studies provide important insights into genomic diversity, there is still limited understanding of the genetic architecture of complex diseases within Ethiopian populations [232].

Current genomic data from Ethiopia provides only limited insight into the genetic architecture of complex diseases across its diverse populations. Available data also suffer from limited sample sizes and sampling bias, typically focusing on only a few major ethnic groups and cities/towns of easily accessible. Consequently, drawing broad conclusions about the genetic makeup or health-related allele frequencies of Ethiopia’s highly diverse population require more thorough investigation taking account broader representation, larger sample sizes and genome-wide approaches to fully capture the complexity of Ethiopia’s genetic diversity and its implications for health and disease.

Furthermore, many studies which were used in this review were relied on candidate gene or single-locus approaches, which, while valuable, do not capture the full spectrum of genome-wide variation. Large-scale, methods such as whole genome sequencing, genome-wide studies, transcriptome-wide studies, and other omics remain underutilized in this context. This lack of comprehensive genomic data limits our ability to draw robust conclusions about disease susceptibility and population genetic landscape.

## Conclusion, future direction, and a call for inclusive genomics research

Ethiopia’s profound genomic diversity is a direct result of millennia of intricate human migration patterns, both outward and return flows, coupled with internal population movements and selective forces from varied environmental adaptations, unique dietary factors, and historical pathogen exposure. These forces have left signatures of positive selection in genes associated with protection against infectious diseases, LP, drug metabolism, diet, UV exposure and adaptation to HA environments. However, not all signals are protective; instead, some variants are linked to increased susceptibility to chronic and infectious diseases. This manuscript underscores Ethiopia’s critical role in human evolutionary history and its unique genomic landscape, which reflects complex patterns of migration, adaptation, and selection. Despite the recognition of Ethiopia’s unique genomic potential and some initial efforts to unravel its genetic architecture, persistent underrepresentation in genomic research limits the translation of these insights into clinical practice. Addressing this gap through inclusive research, capacity building, and global partnership is essential to bridge this gap and for achieving equity in precision medicine and leveraging Ethiopia’s genetic heritage for the benefit of all humanity.

## Data Availability

No datasets were generated or analysed during the current study.

## Abbreviations

ADH: Alcohol dehydrogenase
ADRBK1: Beta-adrenergic receptor kinase 1
AMY1: Salivary amylase gene
APOL1: Apo lipoprotein 1
ARNT2: Aryl hydrocarbon receptor nuclear translocator 2
BHLHE41: HLH family member e41
BMI: Body mass index
BNC2 Gene: Basonuclin zinc finger protein 2 gene
CBARA1: Calcium-binding atopy-related autoantigen 1
CIC: Capicua
CNFN: Cornifelin
CUL3: Cullin3
CXCL17: Chemokine (C-X-C motif) ligand 17
DARC: Duffy antigen receptor for chemokines
DNA: Deoxyribonucleic acid
ESRD: End stage renal disease
FOLR1: Folate receptor alpha 1
FOLR2: Folate receptor alpha 2
G6PD: Glucose-6-phosphate dehydrogenase
GRK2: G Protein-Coupled Receptor Kinase 2
GWAS: Genome wide association study
HA: High altitude
HapMap: Haplotype map
Hb: Hemoglobin
HIF: Hypoxia-inducible factor
HIF-1α: Hypoxia-inducible factor 1-alpha
IFNA: Interferon alpha
IFNL: Interferon lambda
LA: Low altitude
LCT: Lactase gene
LIPE: Lipase E, hormone sensitive type
LP: Lactase persistence
MICU1: Mitochondrial calcium uptake 1
MRC1: Mannose receptor C type 1
5-MTHF: 5 Methyl tetrahydrofolate
mRNA: Messenger Ribonucleic acid
O_2_ sat: Oxygen saturation
*P. falciparum*: *Plasmodium falciparum*
*P. vivax*: *Plasmodium vivax*
PAFAH1B3: Platelet activating factor acetylhydrolase 1beta catalytic subunit 3
PKD2L1: Polycystin 2 like 1, transient receptor potential cation channel
PPARA: Peroxisome proliferator activated receptor alpha
PvDBPII: Duffy binding protein II
SLC24A5: Solute carrier family 24 member 5
SNP: Single nucleotide polymorphism
THRB: Thyroid receptor beta
TRPP3: Transient receptor potential polycystin-3
UV: Ultraviolet
UVRAG: UV radiation resistance associated
VAV3: Vav 3 guanine nucleotide exchange factor
WGS: Whole genome sequence
ZRANB3: Zinc finger RANBP2-type containing 3

## Glossary

Anatomically Modern Human (AMH): Used to distinguish *Homo sapiens* (the only remaining Hominini species) whose anatomical features match those observed in present-day human populations, from now-extinct archaic human species. This classification is particularly useful when anatomically modern and archaic human groups lived alongside each other, such as during the European Paleolithic.
Balancing selection: This is a natural selection process that purposefully keeps genetic variation (multiple alleles) present within a population at higher frequencies than would occur by chance (genetic drift) alone. Rather than eliminating all but the single most advantageous allele, as in directional selection, balancing selection allows different alleles to remain for long periods.
Genetic architecture: Refers to the overall makeup of a quantitative trait or phenotype, encompassing the number of genetic variants involved, the size of their effects, the frequencies at which these variants appear, and how variants interact with each other and with the environment factors that together impact heritability. Many widespread diseases are considered highly polygenic or ‘omnigenic’, indicating that thousands of genetic variants with modest effects may play a role.
Genomic landscape: Describes the full and intricate depiction of all genetic and epigenetic characteristics across an entire genome, often presented in a way similar to a topographical map.
Heritability: A statistical measure estimating how much of the variation in a trait within a population can be attributed to genetic differences. Broad-sense heritability accounts for both additive and dominance effects, while narrow-sense heritability looks specifically at the additive effects from multiple genetic variants.
Hypoxia inducible factor: The Hypoxia-Inducible Factor (HIF) acts as a major transcription factor overseeing the cellular and systemic adaptation to low-oxygen (hypoxic) environments.
Hypoxia: A physiological state where an individual or body part experiences insufficient oxygen levels at the tissue level necessary for regular biological functioning (homeostasis).
Negative (purifying) selection: This concept refers to the evolutionary process where harmful (deleterious) genetic changes are weeded out from a population.
Pharmacogenomics: This scientific discipline explores how the entire genome influences each person’s reactions to medications.
Positive (adaptive) selection: The selection process in which advantageous genetic changes are promoted and quickly increase in frequency throughout a population.
Precision medicine: A developing approach to disease prevention and treatment that adapts health care decisions based on the distinctive genetic makeup, environmental exposures, and life habits of each individual. The objective is to move beyond a generic treatment model and better anticipate which medical strategies will work for given patient groups.
Thrifty gene hypothesis: The Thrifty Gene Hypothesis proposes that genes enabling fat to be stored more efficiently were favored in human ancestors as a way to survive periods of famine. In the modern era with constant access to food, these formerly advantageous genes have adverse effects, increasing rates of obesity and Type 2 Diabetes, though this idea is debated.
